# Secure attachment predicts lower societal cost amongst severely antisocial adolescents

**DOI:** 10.1186/s13034-023-00598-8

**Published:** 2023-05-09

**Authors:** Christian J Bachmann, Sajid Humayun, Madeleine Stevens, Thomas G O’Connor, Stephen Scott

**Affiliations:** 1 Department of Child and Adolescent Psychiatry, University of Ulm, Steinhövelstr. 5, 89075 Ulm, Germany; 2 School of Human Sciences, Faculty of Education, Health and Human Sciences, University of Greenwich, Avery Hill, London, SE9 92UG UK; 3 London School of Economics and Political Science, Houghton Street, London, WC2A 2AE UK; 4 Department of Psychiatry, School of Medicine and Dentistry, University of Rochester Medical Center, 601 Elmwood Ave, Box PSYCH, Rochester, NY 14642 USA; 5 National Academy for Parenting Research, King’s College London, 16 De Crespigny Park, Box 86, London, SE5 8AF UK; 6 Department of Child and Adolescent Psychiatry, Institute of Psychiatry, Psychology & Neuroscience, King’s College London, 16 De Crespigny Park, Box 86, London, SE5 8AF UK

**Keywords:** Antisocial behaviour, Attachment, Callous-unemotional traits, Economic cost, Parenting, Youth

## Abstract

**Background:**

Social and economic costs associated with antisocial behaviour are well-established, but little is known about the potential costs savings/benefits of secure attachment in this high-risk group. We aimed to provide the first test of attachment quality as a distinct predictor of economic costs.

**Methods:**

111 adolescents (10–17 years of age, M = 15.0, SD = 1.6; 71% male) referred to young offender services due to high levels of antisocial behaviour were included. Costs were measured by detailed service-use interview, and attachment security to mother and father elicited through the Child Attachment Interview. The level of antisocial behaviour and callous-unemotional traits were assessed. Cost predictors were calculated using generalised linear models.

**Results:**

Mean 12-months service costs were £5,368 (*sd* 5,769) per adolescent, with justice system and educational service costs being the main components. After adjusting for covariates, economic costs were predicted by attachment quality to fathers, with a difference of £2,655 per year between those with secure (£3,338) versus insecure attachment (£5,993); significant cost effects were not found for attachment quality to mothers. Higher levels of callous-unemotional traits, lower verbal IQ, higher levels of antisocial behaviour, and older age were also significant cost predictors.

**Conclusions:**

Secure attachment to fathers is a predictor of reduced public cost in adolescents with severe antisocial behaviour. This novel finding for severely antisocial youth extends previous findings in less antisocial children and underscores the public health and policy benefits of good caregiving quality and the value of population-level dissemination of evidence-based interventions that improve caregiving quality.

## Introduction

Antisocial behaviour, which is marked by persistent disruptive and aggressive symptoms, and shows a large overlap with the psychiatric concept of “conduct disorder” (CD), is a frequent condition in children and adolescents. Its prevalence is about 5%, and it is often accompanied by psychiatric comorbidity, including attention-deficit/hyperactivity disorder, depression, substance use disorder, and anxiety disorders [1, 2]. Moreover, children and adolescents with antisocial behaviour typically show reduced psychosocial functioning which can lead to poor interpersonal relationships, social exclusion and school dropout; in turn, such an antisocial trajectory carries a greatly increased risk for numerous problems in adulthood, including impaired physical and mental health, difficulties in education, homelessness, drug misuse, criminal offenses, and imprisonment [3–7].

Beyond unfavourable health and psychosocial outcomes, antisocial behaviour also has significant economic consequences, which affect many different sectors of society. High levels of early antisocial behaviour have been shown to be particularly associated with increased public sector justice costs in adulthood [8]. Similarly, longitudinal data from the UK demonstrated that by age 28, public sector costs for children with a diagnosis of CD were nine times higher (mean: 70,019 GBP) than in those without any antisocial behaviour in childhood (mean: 7,423 GBP). In these studies, the majority of costs were associated with criminal activity [9]. Another study estimated that by age 20, mean costs associated with criminal convictions are about 500,000 USD, with the majority of costs occurring during mid-to-late adolescence, and antisocial behaviour being one of the strongest risk factors [10].

In youths with antisocial behaviour, older age, male sex, higher levels of antisocial behaviour, and lower socio-economic status have so far been identified as risk factors for higher costs at follow-up [9, 11, 12]. Recently, attachment insecurity and low parental sensitivity have also been shown to predict increased cost in children exhibiting only moderate levels of antisocial behaviour [13, 14]. These recent findings are important for several reasons. Most notably, whereas most predictors of cost are associated with child or adolescent personality traits or social conditions that may not be readily amendable to intervention, attachment quality, as an outcome of early caregiving quality, is a common and highly plausible intervention target. Relatedly, whereas previous research on economic costs has focused on risks for greater costs, a focus on (secure) attachment quality could potentially identify, more directly, sources of cost savings.

The aim of this study was to extend prior research by testing the hypothesis that attachment quality of adolescents to their parents might predict economic costs for society in a high-risk sample of adolescents who were already showing substantial levels of antisocial behaviour.

## Methods

### Participants

We used data from the Study of Adolescents’ Family Experiences (SAFE), a randomised controlled trial of Functional Family Therapy (FFT), which was carried out in the UK from 2008 to 2011 [15]. Participants of the SAFE trial were 111 adolescents (10–17 years of age (mean: 15.0 (SD = 1.63) years); 71% male), who had been recruited through Youth Offending Services, Targeted Youth Support Services, and other crime prevention agencies in two English counties. All participants had been sentenced for offending or were receiving agency intervention following contact with the police for antisocial behaviour. The adolescents and their families were allocated to either Functional Family Therapy (FFT) plus Management As Usual (MAU) (N = 65), or to MAU alone (N = 46). In addition to recording socio-demographic data, clinical and cost data (from a societal perspective; excluding cost of the RCT intervention) were recorded for the 6 months prior to randomization (baseline), and for the 6 months after randomization (6 months follow-up). Assessments included interviews and questionnaires of parenting behaviours, youth antisocial behaviour, IQ, conduct disorder, and adolescent antisocial psychopathy.

### Measures

#### Family characteristics

A structured interview with the primary caregiver assessed details about family structure and income, ethnicity and parental education.

#### Conduct disorder symptoms

CD and Oppositional-Defiant Disorder (ODD) symptoms according to the Diagnostic and Statistical Manual of Mental Disorders, Fourth Edition, Text Revision (DSM-IV-TR) were assessed using the *Adolescent Parent Account of Child Symptoms* (APACS), a semi-structured diagnostic interview administered to parents [16]. The mean single-measure ICC reliability on 20 randomly selected cases for ODD and CD criteria was 0.95, for ODD symptom count 0.99 and for CD symptom count 0.98 [15].

#### Antisocial behaviour

Antisocial acts were reported by the young people using the *Self-Report Delinquency (SRD)* questionnaire [17]. This consists of 19 items covering a range of antisocial acts divided into three scales (home problems, school misbehaviour, substance abuse). The SRD has good psychometric properties (internal consistency in the SAFE sample: α = 0.87 [15]) and correlates substantially with official police arrests [18].

#### Callous-unemotional (CU) traits

These were assessed using parent reports from the “callous-unemotional traits” subscale of the Antisocial Process Screening Device (APSD) [19]. The APSD is a well-validated instrument for the screening of adolescent psychopathy [20].

#### Cognitive ability

Participants’ IQ was assessed by a trained examiner using the *Wechsler Abbreviated Scale of Intelligence (WASI)* [21].

#### Attachment security

Adolescents’ attachment security was assessed using the *Child Attachment Interview (CAI)* [22]. The CAI is a well-validated, semi-structured interview designed to elicit young people’s mental representations of their parental attachment figures through asking them a series of questions about specific experiences of caregiving; it has been applied in diverse clinical settings. Responses were coded according to a manual, and ratings were made separately for each parent. Attachment data could be assessed for 103 mothers, and for 75 fathers. The higher rate of missing data on fathers was due to the adolescent having had no contact with the father for several years, or their being uncontactable, an approach previously validated for this instrument [22]. For the purpose of this study, the Secure versus Insecure designation was used. Two coders were trained by the instrument developers, and the reliability on 20 training cases for the Secure–Insecure split was 90% agreement (ϰ=0.79). The coders were blind to other data collected on the youths and did not conduct the interviews.

#### Parenting style

Parents completed the short version of the Alabama Parenting Questionnaire, the APQ-15. The 15 items are classified into five domains: Involvement, Positive Parenting, Poor Monitoring/Supervision, Inconsistent Discipline, and Corporal Punishment. The APQ-15 has good reliability and validity [23], and the internal consistency in this sample was α = 0.74 [15].

#### Service use and costs

Societal costs for a period of 12 months were calculated using the Client Service Receipt Inventory (CSRI) [24]. The CSRI is a well-established semi-structured interview where parents are asked about health, educational and social care services used by their child, or by family members related to the child’s behaviour over a specified time period. Costs for each type of service use were then calculated based on unit costs at 2010 prices (Appendix 1). The unit costs were taken from official sources where possible [25, 26] or else from a compilation [27]. The unit costs (per appointment, per contact) were multiplied by frequency and duration of service use for each agency. Where data on the length of the contact was missing we used typical contact lengths where these were available, or as taken from the study data. Where necessary we assumed 30-minute appointments, except for ‘talking therapies’ (family therapist, psychologist, counsellor and social worker) where we assumed one hour per contact.

### Statistical analysis

All statistical analyses were performed using SPSS 27.0. Total cost was the dependent variable. Due to the left-skewness of the cost data, a Tweedie distribution was assumed and data were analysed using generalised linear models which do not assume a normal distribution [28]. Based on previous literature and a priori assumptions, several covariates were included: maternal education and eligibility for free school meals to indicate socio-economic status; youth sex, age and IQ; antisocial behaviour level (self-report); CU traits indexed from APSD parent reports. Treatment arm of the underlying study was included as a covariate in analyses to examine if attachment security predicted independently of treatment condition and other covariates. Separate analyses were conducted for attachment to mother and father. For the comparison of means, Mann-Whitney-U tests were used.

## Results

Table 1 shows the characteristics of the sample. 71% of youths were male; more than half of the sample were eligible for free school meals (as a proxy for low family income), and their mean IQ at 84 was nearly one SD below average. Nearly two-thirds of mothers had left school by 16 years (60%, vs. national norm 18%), and more than 50% of families were single-parent families.

**Table 1:** Demographic and clinical characteristics of participants

Characteristic	SAFE sample (N = 111)	National norms/ Unaffected sample
Adolescent age in years (mean, SD)	15.0 (1.6)	---
Adolescent male	71% (79/111)	51% [49]
Adolescent ethnic minority	10% (11/111)	11% [49]
Adolescent Full IQ (mean, range)	84 (range: 56–116)	100 [49]
Family structure (single parent)	55% (61/111)	32% [49]
Maternal education (left school by age 16)	60% (67/111)	18% [49]
Free school meals	52% (56/108)	17% [49]
Antisocial behaviour level (self-reported delinquency) (mean, SD)	61.5 (35.3)	2.6 (3.7) [34]
Callous-unemotional traits (mean, SD)	5.8 (2.3)	2.4 (2.1) [50]
Secure attachment to mother	40% (41/103)	68% [41]
Secure attachment to father	23% (17/75)	55% [41]

The rate of self-reported delinquent acts was very high, as were CU personality traits. Rates of attachment security (both to mothers and to fathers) were significantly lower than in normal risk samples.

Table 2 provides bivariate associations between variables. Total cost was significantly correlated with severity of antisocial behaviour, treatment arm of the original trial, and lower verbal IQ; higher levels of antisocial behaviour showed a significant correlation with CU traits, poor parental monitoring, and eligibility for free school meals. There was substantial agreement in adolescents’ attachment to mothers and fathers; of the 29 adolescents who were classified as having a Secure attachment to mother, 13 were classified as having a Secure attachment to father. Inversely, of the 17 adolescents who were classified as having a Secure attachment to father, 13 were classified as having a Secure attachment to mother.

**Table 2:** Correlation between measures (Spearman’s rho)

	Treatment arm	Age	Sex	Ethnicity	Verbal IQ	Maternal education	Free school meals	Family structure	Poor monitoring	Antisocial behaviour	CU traits	Attachment to mother	Attachment to father
Age	− 0.023												
Sex	− 0.011	− 0.003											
Ethnicity	0.096	0.012	− 0.153										
Verbal IQ	− 0.127	0.078	− 0.122	0.072									
Maternal education	− 0.103	− 0.014	0.069	0.088	0.479**								
Free school meals	− 0.025	− 0.269**	0.003	− 0.142	− 0.338**	− 0.052							
Family structure	0.015	− 0.060	− 0.010	− 0.075	− 0.183	− 0.012	0.358**						
Poor monitoring	0.090	0.037	− 0.113	0.057	0.028	0.012	0.170	0.105					
Antisocial behaviour	0.126	− 0.016	0.188	− 0.097	0.169	0.134	− 0.245*	− 0.121	− 0.453**				
CU traits	0.051	− 0.071	− 0.051	− 0.046	0.025	0.003	− 0.139	− 0.136	− 0.270**	0.305**			
Attachment to mother	− 0.150	− 0.136	0.127	− 0.003	− 0.113	− 0.106	− 0.030	0.095	0.007	− 0.064	0.120		
Attachment to father	0.028	− 0.159	0.159	0.010	− 0.218	− 0.008	0.000	0.055	− 0.109	0.028	0.170	0.420**	
Total cost	0.208*	0.152	0.167	− 0.167	− 0.256**	− 0.103	− 0.056	0.183	− 0.084	0.263**	0.008	− 0.061	0.209

Annotation: *: correlation significant at p < 0.05; **: correlation significant at p < 0.*01*

Table 3 shows costs according to attachment security to mother and father without considering covariates. Adolescents who were securely attached to their fathers cost £3,338 per year, whereas those insecurely attached cost £5,993 per year. Regarding mothers, securely attached youths cost £5,315 per year, whereas insecurely attached youths cost £5,380 per year. The largest cost component were costs for the justice system, followed by education services, and by social care services.

**Table 3:** Cost domains (in £) per individual, by attachment security to mother and father

Cost type		Total sample (N = 111)	Attachment to mother			Attachment to father			
			Secure (N = 41)	Insecure (N = 62)	p	Secure (N = 17)	Insecure (N = 58)	p	
**Total costs**	**Mean**	**5,368**	**5,315**	**5,380**	0.535	**3,338**	**5,993**	0.072	
	Median	3,333	4,202	3,107		2,386	3,425		
	Maximum	30,121	16,212	30,121		16,212	30,121		
**Justice system costs** (young offender support, youth justice)	**Mean**	**3,157**	**3,645**	**3,059**	0.346	**2,261**	**3,637**	0.591	
	Median	1,128	1,188	1,270		944	1,244		
	Maximum	27,778	15,399	27,778		15,399	27,778		
**Education services costs** (educational support, behaviour support at school)	**Mean**	**1,341**	**1,047**	**1,527**	0.844	**584**	**1,605**	0.057	
	Median	418	443	326		75	568		
	Maximum	19,692	5,944	19,692		2,890	19,692		
**Social care services costs**	**Mean**	**425**	**247**	**323**	0.394	**80**	**295**	0.049	
	Median	0	0	10		0	10		
	Maximum	16,176	1,911	2,139		735	2,139		
**Health services costs** (primary care, hospital, mental health services)	**Mean**	**239**	**227**	**262**	0.396	**372**	**241**	0.934	
	Median	92	48	111		124	86		
	Maximum	3,739	3,739	1,852		3,739	1,852		
**Costs for family members** (primary caregiver services, other relative services)	**Mean**	**206**	**149**	**208**	0.577	**41**	**215**	0.186	
	Median	0	0	0		0	0		
	Maximum	3,820	1,492	3,820		417	3,820		

*Annotation: p = significance value for difference in medians by Mann-Whitney U test*

A generalised linear model was carried out to determine whether or not the difference in cost between securely and insecurely attached youth was significant after controlling for covariates (Table 4). Attachment insecurity to fathers predicted highly significant increased cost (p = 0.001), as did CU traits. Further predictors of cost were older age, youth with a lower IQ, and those with higher delinquency level. None of the other covariates (family structure, parental monitoring, parental educational attainment, youth ethnicity) was significantly associated with cost and including them in the model did not substantively alter the prediction from attachment security to father (data not shown).

**Table 4:** Predictors of costs (generalised linear model)

	Attachment to mother (N = 98)		Attachment to father (N = 70)	
Predictor	Wald Chi Square	p	Wald Chi Square	p
Treatment arm	1.102	0.294	0.061	0.805
Adolescent age	3.762	0.052	14.111	< 0.001
Adolescent male sex	1.350	0.245	0.448	0.503
Adolescent verbal IQ	7.272	0.007	7.895	0.005
Free school meals	1.385	0.293	3.369	0.066
Antisocial behaviour level	7.544	0.006	11.498	< 0.001
Callous-unemotional traits	3.315	0.069	10.666	0.001
Attachment to father			8.953	0.003
Attachment to mother	0.056	0.813		

An additional analysis was carried out on the subset of families in which we had attachment quality data for both mothers and fathers (Table 5); secure attachment to both parents was used as a predictor. Results indicated that secure attachment to both parents predicted further cost savings than to father alone (p = 0.001; Table 5).

**Table 5:** Predictors of costs (generalised linear model), including attachment to both parents (N = 70)

Predictor	Wald Chi Square	p
Treatment arm	0.003	0.957
Adolescent age	14.191	< 0.001
Adolescent male sex	0.681	0.409
Adolescent verbal IQ	7.424	0.006
Free school meals	2.203	0.138
Antisocial behaviour level	14.350	< 0.001
Callous-unemotional traits	13.767	< 0.001
Secure Attachment to both parents	10.492	0.001

## Discussion

This study analysed predictors of societal cost in a sample of adolescents, all of whom were severely antisocial and had come into contact with agencies dealing with young delinquents. We found that secure attachment to father was associated with significant reduction in costs in this very high-risk adolescent sample. Whilst attachment security to mother was not a predictor of cost on its own, lack of a secure attachment to both parents was associated with increased costs to society. The cost benefit of a secure attachment was independent of other factors, including other significant predictors of cost, i.e. higher levels of CU traits, more severe antisocial behaviour, lower IQ and older age. This is the first demonstration of the economic benefits of a secure attachment, a modifiable risk, in a very high-risk sample of adolescents.

The reduction in costs attributable to secure attachment, in descending order of amount, were incurred through less involvement with the justice system, less extra educational provision, and reduced need for social care services, health services and fewer personal costs to the family. The same order of costs was reported by Scott et al. [9], who studied public sector costs of by 142 10-year-old children with antisocial behaviour who were then followed up to age 28 years. The important observation from these analyses is the broad-based costs that constitute the economic benefits implied by a secure attachment in the current study.

To our knowledge, this is the first paper to show that attachment security predicts reduced cost to society within a sample with high levels of antisocial behaviour. A previous paper from our team has shown that attachment security predicts costs in children with less severe levels of antisocial behaviour, where security to the father was also found to be more strongly predictive of costs than was secure attachment with mother [14]. It is therefore noteworthy that the same processes hold true at the extremes of the distribution of antisocial behaviour, particularly given the popular and scientific presumptions about the difficulty of improving the life chances of this population. Furthermore, the prediction of costs from attachment security was independent of three factors that are well known to be associated with increased offending – older age, higher level of antisocial behaviour, and lower verbal IQ [9, 12, 29–32]. Intervention studies commonly target attachment quality, including in adolescence [33], and there is now evidence that even children with a history of severe abuse and neglect can form secure attachments in adolescence [34]. Collectively, this evidence suggests the modifiability of attachment security and the possibility that attachment-based interventions may yield behavioural and economic benefits. It is exceedingly rare for observational studies and uncommon in interventional studies of caregiving quality to include formal cost analyses. Our findings suggest that including cost analyses may offer substantial opportunities to place caregiving and parenting studies in a broader public health and economic context. Moreover, such an approach might yield policy-oriented evidence to support parenting programmes like e.g. The Incredible Years, or Parent-Child Interaction Therapy (PCIT), for which there is sufficient evidence regarding their effectiveness in improving both parenting and children’s behavioural outcomes [35–38].

The underlying mechanism linking attachment security to father and reduced costs is not clear. It could, for example, reflect internalisation of adaptive behaviours and cognitions and emotional regulation strategies from fathers who are experienced as emotionally available and supportive [39]. There is some evidence that secure paternal attachment may improve emotional regulation abilities [40], which in turn might lead to more resilience and better coping strategies, thus reducing the need of support by justice or education system services. The findings might also be related to poorer monitoring by the father which may accompany insecure attachment [41]. Attachment insecurity is known to be associated with a wide range of poorer social, emotional and behavioural outcomes in children and adolescents [40, 41]. The current study on economic costs is part of a growing set of studies that assess the benefits of Secure attachment – as a reflection of caregiving quality – that extend beyond traditional bounds of psychological and behavioural health to physical, occupational, and social health and well-being [42].

Although not directly demonstrated in this report, the quality of the parenting environment is a crucial determinant of attachment security, with sensitive responding being particularly implicated. This association is not just true for infancy and early childhood, but also holds in adolescence, so is likely to be important here [43]. Moreover, longitudinal studies suggest that less sensitive responding in childhood is associated with greater financial cost to society in adolescence [13]. The implication is that economic costs of parenting likely extend to attachment-based and sensitive parenting-based models and methods. In this context, it is notable that economic costs were not reliably associated with measures from a widely-used parenting questionnaire (APQ-15), suggesting that not all dimensions and methods of measuring parenting may be associated with economic costs. Detecting cost benefits of parenting quality may require the kind of clinically-sensitive and time-intensive approach that was used in the current study.

This is also the first paper to report that CU traits are associated with higher costs, even after accounting for level of delinquency and other covariates, including IQ and socio-economic condition. CU traits are associated with less empathy, more offending, higher teacher/student conflict [44], less concern about school performance, less remorse, and poorer treatment response [45]. It may be that the prediction from CU traits is not simply a reflection of severity but of type of severe disturbance. The higher costs associated with CU traits may also reflect the possibility that these adolescents may be more likely to come to the attention of authorities [46, 47], evoke increased likelihood of intervention, and have a higher need for ongoing support in various domains of their life [48], which in turn would lead to higher costs.

### Strengths and limitations

This study has several strengths: The sample was characterised in terms of socio-demographic and clinical characteristics, and these characteristics were assessed using a multi-method, multi-informant approach, which included investigator ratings from semi-structured parent interviews, an extensive service use interview, youth self-reports, psychometric assessments, and blinded coding of attachment security. Furthermore, in the statistical analyses considerable adjustments were made for potential confounders. The credibility of the findings is increased by previous studies finding father attachment to be an important predictor of cost in less severe samples [14], and follow-up studies showing broadly the same distribution of agencies involved in extra costs [9].

In terms of limitations, this study employed a cross-sectional design, which prohibits causal attributions between costs and predictors. Also, because of the nature of the sample, with many adolescents coming from non-intact families, attachment data were not available for mothers and fathers for all adolescents. Additionally, the study population was very high-risk and already in the social care system in the UK; the findings and costs obtained in this study may not generalise to other samples and settings.

## Conclusions

The study indicated that attachment security in adolescence remains an important predictor of costs to society in a notably antisocial sample. Likewise, CU traits increased costs over and above the level of antisocial behaviour. Both of these characteristics are amenable to evidence-based parenting programmes delivered in childhood. Wider provision of programmes to support parental sensitivity and child attachment quality may improve the well-being of the individual child and their family and save money for society.

## Data Availability

The data that support the findings of this study are available from the senior author, SS, upon reasonable request.

## Appendix 1: Unit cost for each service at 2009–2010 prices

**Table:**

Service	Unit Cost	Notes
**Justice system**		
**Young offender support**		
YOT case worker	£131/visit	
**Youth justice**		
Reprimand &/or final warning	£188/case	£389 when including police cell
Lawyer	£114/hour	
Attendance centre	£27.4/visit	
Police station	£24/visit	
Court appearance	£480/appearance	
Police cell-nights	£352/night	
Youth custody-nights	£223/night	
Prison-nights	£79/night	
Electronic surveillance tag	£2,536/tag	
**Education services**		
**Educational support**		
Smaller group lessons	£5/hour	
Classroom assistant	£16/hour	
Individual tuition	£52/hour	
School mentoring	£49/hour	
After school club	£3/hour	
Home-school liason	£121/hour	
Extra home tuition	£78/hour	
School doctor	£32/consult	
School nurse	£10/consultation	
Connexions advisor	£49.5/hour	0.5 h estimated time
**Behaviour support**		
Behaviour management class	£9/hour	
Key worker-school	£73.5/consultation	
Psychiatrist	£156/hour	
Psychologist	£80/hour	
Educational social worker	£50/consult	
Educational psychologist	£54/hour	
School counsellor	£49/hour	
**Health services**		
**Primary health care**		
GP	£32/consultation	
GP nurse	£18.5/hour	0.5 h estimated time
Repeat prescription	£8.8/prescription	
Other community nurse	£24/consult	
**Hospital**		
Hospital inpatient	£447/bed day	
Outpatient clinic	£149/visit	
A&E or Minor Injuries Unit	£76/treatment	
Specialist doctor	£71/hour	
**Mental health services**		
Family therapist	£69/hour	
Psychiatrist	£156/hour	
Psychologist	£80/hour	
Psychiatric nurse	£24/hour	
Counsellor	£44/consultation	
**Social care services**		
Social worker	£147/hour	
Key worker	£147/hour	
**Family (primary caregiver, other relatives)**		
GP	£32/consultation	
Practice nurse	£10/consultation	
Hospital outpatient	£152/visit	
Counsellor	£44/consultation	
Alternative therapy	£41/hour	
Self-help/support group	£8/session	
Phone helpline	£13/consultation	
Parenting programme	£98/visit	

*Unit cost sources: Education.gov.uk;* *Beecham J, Bauer A, Stevens M. EPP Unit Costs, Working paper 5v2. not publicly available; 2011;* *Curtis L. Unit Costs of Health and Social Care 2010. Canterbury Personal Social Services Research Unit, University of Kent; 2010.*

*Where supports were reported over a longer period than that requested on the questionnaire, these have been adjusted to reflect the one-year period. Where a service has been used but there is no response on the number of contacts, we assume one contact only.*

## Abbreviations

APACS: Adolescent Parent Account of Child Symptoms
APQ-15: Alabama Parenting Questionnaire, short version
APSD: Antisocial Process Screening Device
CAI: Child Attachment Interview
CD: Conduct Disorder
CU: Callous-unemotional
CSRI: Client Service Receipt Inventory
DSM-IV-TR: Diagnostic and Statistical Manual of Mental Disorders, Fourth Edition, Text Revision
FFT: Functional Family Therapy
ICC: Intraclass Correlation Coefficient
MAU: Management As Usual
ODD: Oppositional-Defiant Disorder
SAFE: Study of Adolescents’ Family Experiences
SD: Standard Deviation
SPSS: Statistical Package für Social Sciences
SRD: Self-Report Delinquency
UK: United Kingdom of Great Britain and Northern Ireland
WASI: Wechsler Abbreviated Scale of Intelligence
